# Using spatial distribution modeling of commercial species to inform management of small‐scale fisheries in a Mediterranean marine protected area

**DOI:** 10.1002/ece3.70169

**Published:** 2024-09-13

**Authors:** G. La Manna, F. Ronchetti, M. Moro Merella, R. Vargiu, F. Perretti, G. Ceccherelli

**Affiliations:** ^1^ Dipartimento di Scienze Chimiche Fisiche Matematiche e Naturali Università di Sassari Sassari Italy; ^2^ National Biodiversity Future Center Palermo Italy; ^3^ MareTerra – Environmental Research and Conservation Alghero Italy

**Keywords:** artisanal fishery, fish, fishing closure area, habitat suitability, MaxEnt

## Abstract

Marine protected areas (MPAs) make an essential contribution to the spatial management of critical areas, the conservation of coastal species exploited by human activities, and the sustainable use of marine resources. Within MPAs, fishing closure areas are among the most used small‐scale fishery management tools, even though their effectiveness largely remains untested or controversial. To reduce the impact of small‐scale fisheries on marine resources, a seasonal fishing closure area (SFCA) was established beginning in 2022 in autumn–winter season inside the Capo Caccia–Isola Piana MPA (Sardinia, northwestern Mediterranean Sea). Here, we assessed a posteriori whether the areas of higher habitat suitability for eight species/taxa of relevant ecological value and economic interest to small‐scale fisheries were included in the established SFCA, adequately meeting the ecological objectives of the MPA. Thus, landing data (from 2019 to 2023) were used as occurrence records to develop MaxEnt distribution models for the eight target species/taxa. The model outputs allow us to draw important insights about the spatial adequacy of the SFCA established within the MPA aimed to protect the most exploited marine resources. Furthermore, the modeling exercises were useful for understanding the local processes influencing species' habitat selection and to identify essential areas for the target species that could remain unrevealed in larger‐scale investigations.

## Graphical abstract

A modeling approach was used to investigate the spatial adequacy of a seasonal fishing closure area established within a Mediterranean MPA. The local ecological processes influencing fish habitat selection were identified. Insights for small‐scale fishery management were found.

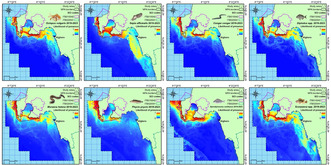

## 1 INTRODUCTION

Marine protected areas (MPAs) make an essential contribution to the spatial management of critical areas and conservation of coastal species exploited by human activities (Grorud‐Colvert et al., 2021). Since their introduction at the end of 19th century (Claudet, 2011), MPAs have progressively become a widely common tool for managing marine resources. Within MPAs, fishing or gear closure areas are among the most used fishery management tools, even though their effectiveness largely remains untested (Kaiser, 2011) or controversial (FAO, 2011; Garcia et al., 2013). Within MPAs, fishing closure areas have the potential to protect priority habitats and spawning grounds and reduce the exploitation of sedentary fish species, especially when management is based on species distribution and habitat use data availability (Weigel et al., 2014). Concurrently, fishing closure areas can also have immediate high costs for the local fisher communities, while benefits to fisheries in terms of increase in fish biomass and recruitment or spillover effect may occur in the long‐term (Topor et al., 2019) and may not be immediately perceived by local fishers (Garcia et al., 2013). Furthermore, since fishing closure areas have been often designed for conservation purposes and using scant ecological‐based criteria (Harmelin, 2000), rather than being implemented by informed actions based on the potential consequences for both the fisheries and ecosystem, their establishment often leads to conflict between human demand and biodiversity conservation.

In Europe, the fishery sector is still highly relevant despite the progressive decline. Namely 82% of all fishing vessels in the EU Mediterranean countries belong to artisanal fishers (small‐scale fisheries), providing around 111,000 jobs (FAO, 2023). This traditional fishery is conducted mainly between spring and autumn in coastal areas (within 12 nautical miles from the coast) easily reachable in a short time and by small vessels (less than 12 m in length; Raicevich et al., 2018); moreover, in the Mediterranean Sea, it is generally practiced by family members, who often obtain an income on the edge of the poverty threshold (La Manna et al., 2024). Thus, deciding the spatial location of fishing closure areas is a critical step in the management of small‐scale fisheries inside MPAs, both from a conservation and socio‐economic point of view: a balance between the ecological benefits obtained by the fishing closure area and the social costs due to the fishing effort displacement must be found. The availability of spatial information (e.g., species distribution) is of paramount importance to draw effective conservation planning (Margules & Pressey, 2000; Welch et al., 2019). For example, a fishing closure area may be several times larger than the home range of the target species it should protect or, otherwise, may not contain sufficient suitable habitat for them (Abecasis et al., 2014), thus representing a cost for fishers without offering adequate protection to be effective. In this context, species distribution models (SDMs, also known as habitat suitability or ecological niche models, Guisan et al., 2013), which use spatially georeferenced data to identify the relation between species occurrence with biotic and abiotic variables, are particularly useful to obtain ecological insights (Elith & Leathwick, 2009) such as identifying species habitat, special areas of interest for biodiversity (Franklin & Miller, 2010; Guisan et al., 2013), and human activities (La Manna et al., 2023; Navarro et al., 2015), and thus inform local or regional coastal planning and policymakers (Chamberlain et al., 2023).

In the framework of fishery management, in the absence of high‐quality data coming from independent surveys and research programs, occurrence records can be obtained from fishery‐dependent data. These data usually come from scientific observers measuring catches on board of commercial fishing vessels or during the landing operations, or from logbooks required by local, national, or international regulations; they may represent the only commercial species distribution data available (Karp et al., 2022), also in the context of MPAs management. These data can be of poor quality compared to fishery‐independent data (Conn et al., 2017; Thorson et al., 2020) since (i) they are not taken following a sampling design; (ii) specimens can be identified to taxonomic levels higher than species; (iii) catches can come from different vessels and gears; and (iv) sampling effort is often unknown. However, recent studies show that, if used appropriately, they can result in adequate distribution predictions (Crear et al., 2021; Ducharme‐Barth et al., 2022; Pennino et al., 2016). Particularly, some SDM approaches can be used to address bias related to data limitations, for example, the maximum entropy algorithm (MaxEnt), one of the best performing (Hernandez et al., 2006; Valavi et al., 2022) and most widely used methods to model fish distribution (Melo‐Merino et al., 2020). Thus, in the present study, landing data were used as occurrence records to develop MaxEnt distribution models for species/taxa of relevant ecological value and economic interest to small‐scale fisheries in Alghero (Sardinia, northwestern Mediterranean Sea). To reduce the impact of small‐scale fisheries on marine resources, a seasonal fishing closure area (SFCA) was established in 2022 in autumn–winter season inside the nearby MPA (Capo Caccia–Isola Piana MPA). In this study, the model outputs were used a posteriori to assess whether, for the target species, the areas of higher habitat suitability were included in the seasonal fishing closure area, adequately meeting the ecological objectives of the MPA.

## 2 METHODS

### 2.1 Study area

**FIGURE 1:**
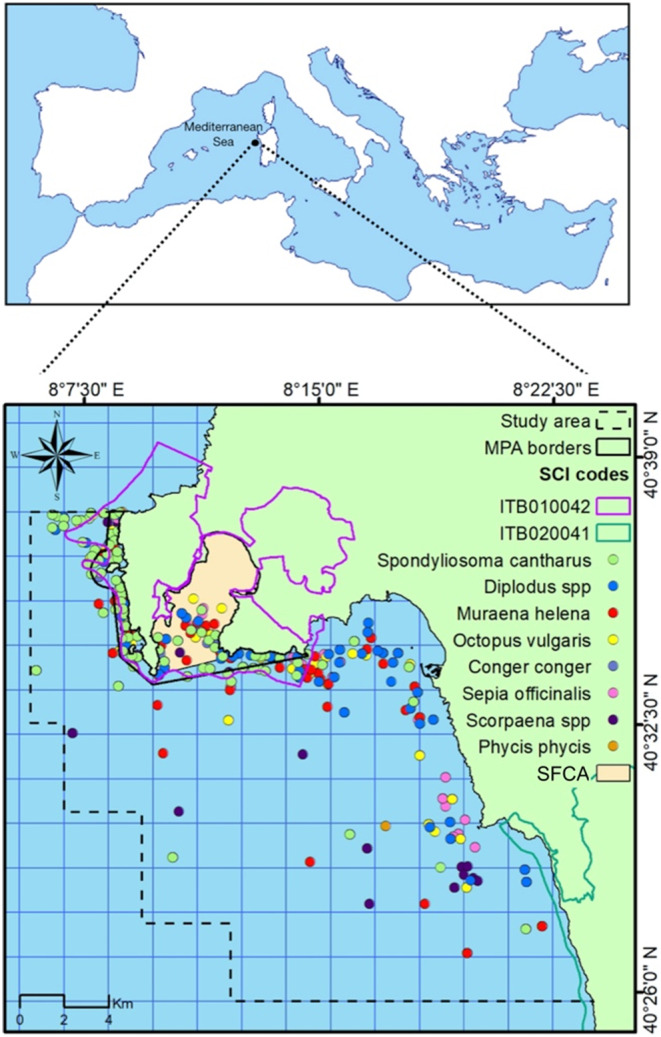
Study area. Colored points: Occurrence records per target species used in the modeling exercises. Dotted line: Borders of the study area; solid line: MPA borders; purple and green lines: SCIs boundaries; pale pink area: Seasonal fishing closure area (SFCA). The figure was created by the authors using QGIS.

The marine protected area Capo Caccia–Isola Piana (MPA) is located on the northwestern coast of Sardinia (Italy) within the municipality of Alghero (40°34′ N, 8°13′ E; Figure 1). The MPA was established in 2002 to protect an area of significant naturalistic and geomorphological interest, with particular regard to the ecological, cultural, educational, and economic importance of the marine and coastal habitat, flora and fauna, such as the endemic Mediterranean seagrass (*Posidonia oceanica*) and coralligenous reef, the red coral (*Corallum rubrium*), the ribbed Mediterranean limpet (*Patella ferruginea*), the noble pen shell (*Pinna nobilis*), and the common bottlenose dolphin (*Tursiops truncatus*). For its scientific, esthetic, and cultural values, the MPA was also declared SPAMI (Specially Protected Areas of Mediterranean Importance) in 2009 by the Contracting Parties of the Barcelona Convention.

The MPA extends for 26.31 km^2^, along 36 km of coastline, and is divided into: two A zones (full protection: 0.38 km^2^) where all activities are forbidden except for scientific research; two B zones (general protection: 5.47 km^2^); and one C zone (partial protection: 20.46 km^2^) where scuba diving, boat navigation at regulated speeds (between 5 and 8 knots), guided visits, anchoring and mooring in identified areas, and recreational and small‐scale fishing and fishing tourism are permitted but regulated by the management body.

### 2.2 Fishing regulation

Within the MPA, small‐scale fishing is reserved for resident fishers authorized by the management body, and it is allowed only with selective gears: pots, gillnets, and trammel nets. Longlines are allowed but rarely used. The maximum length of trammel net and gillnet per fishing vessel was fixed to 2000 m, while the maximum number of pots that each vessel could use was limited to 100. Furthermore, it is forbidden to use more than one type of fishing system simultaneously. In 2022, an SFCA (from September to March) inside the C zone of the MPA was established, where neither professional nor recreational fishing is permitted (Figure 1).

### 2.3 Data collection and target species

Small‐scale fishery landing data were obtained from the systematic MPA fishery monitoring program, which quantified the landings (in terms of species composition, individual length, and biomass) from March to October 2019, 2020, 2021, 2022, and 2023 during 31, 46, 71, 79, and 161 fishing operations, respectively, from the 21 vessels permitted to fish inside the MPA. Data related to fishing site (geographic coordinates collected by the GPS of the fishing vessels), haul duration, and gear features (net length and mesh size, number and size of pots) have also been recorded.

From the preliminary exploration of these data, eight species/taxa were selected on the basis of the number of available occurrence records (fishing operations where each species was present) and the ecological and economic relevance: *Octopus vulgaris*, *Sepia officinalis*, *Conger conger*, *Muraena helena*, *Phycis phycis*, *Spondyliosoma cantharus*, *Diplodus* spp., and *Scorpaena* spp. Many of these species occupy the same habitat type, thus an overlapping distribution was expected (Table 1).

**TABLE 1:** Ecology and number of occurrence records per species/taxa and year. MaxEnt models were run only when the number of records per year was >15. In brackets is the number of records before filtering procedure used to cope with the spatial autocorrelation of occurrence records.

Species/taxa	Ecology		Year						
	Favored habitats	Favored prey		2019	2020	2021	2022	2023	2019–2023
*Octopus vulgaris*	Seagrasses and coral reefs from the surface up to about 100 m in depth Jereb and Roper (2005)	Benthic macrofauna (mainly gastropods and bivalves) Jereb & Roper (2005)	No. of records	14 (18)	14 (22)	26 (28)	21 (25)	52 (71)	103 (164)
			MaxEnt models	No	No	Yes	Yes	Yes	Yes
*Sepia officinalis*	Sandy bottoms and shallow coral reefs (generally up to 50 m) Jereb and Roper (2005)	Fish and crustaceans Jereb and Roper (2005)	No. of records	4 (5)	10 (11)	15 (15)	4 (4)	28 (29)	54 (64)
			MaxEnt models	No	No	No	No	No	Yes
*Diplodus* spp.	Coastal habitat (rocky reef and seagrass bed), generally up to 50 m in depth Göthel (1992)	Benthic macrofauna (crustaceans, worms, and mollusks) Göthel (1992)	No. of records	19 (36)	20 (39)	21 (29)	18 (24)	55 (96)	99 (224)
			MaxEnt models	Yes	Yes	Yes	Yes	Yes	Yes
*Phycis phycis*	Hard and sandy–muddy bottoms near rocks	Small benthic fish and various invertebrates Cohen et al. (1990)	No. of records	10 (13)	11 (17)	28 (40)	26 (39)	26 (30)	79 (139)
			MaxEnt models	No	No	Yes	Yes	Yes	Yes
*Conger conger*	Coastal rocky and sandy bottom during the juvenile stage, and deeper waters (up to 500 m) upon reaching adulthood Bauchot and Saldanha (1986)	Fish, crustaceans, and cephalopods Bauchot and Saldanha (1986)	No. of records	8 (9)	12 (17)	21 (25)	18 (22)	29 (30)	74 (103)
			MaxEnt models	No	No	Yes	Yes	Yes	Yes
*Scorpaena* spp.	Rocky bottoms or seagrass beds, from a few up to hundreds of meters in depth Göthel (1992)	Benthic fish, crustaceans, and mollusks Göthel (1992)	No. of records	15 (36)	19 (62)	28 (40)	30 (53)	63 (154)	123 (345)
			MaxEnt models	Yes	Yes	Yes	Yes	Yes	Yes
*Muraena helena*	Highly territorial species, found in rocky bottoms and coral reefs, from a few up to hundreds of meters in depth Smith and Böhlke (1990)	Fish, crabs, and squid Smith and Böhlke (1990)	No. of records	8 (8)	9 (14)	17 (18)	9 (12)	48 (302)	77 (354)
			MaxEnt models	No	No	Yes	No	Yes	Yes
*Spondyliosoma cantharus*	Seagrass beds, rocky, and sandy bottoms from a few up to about 300 m Bauchot and Hureau (1990)	Seaweeds and small invertebrates Bauchot and Hureau (1990)	No. of records	12 (16)	17 (26)	28 (42)	24 (31)	26 (30)	82 (145)
			MaxEnt models	No	Yes	Yes	Yes	Yes	Yes

Since the study area is relatively small in size and data are fishery dependent, to cope with the spatial autocorrelation of occurrence records (e.g., multiple occurrences in relatively close spatial proximity), for each species, the repeated records within a buffer of 250 m radius were removed using “Spatially Rarefy Occurrence Data for SDMs” tool in the SDM toolbox 2.5 (Brown et al., 2017). The tool works by filtering location data by a user‐defined distance, condensing occurrence points to a single point within the specified Euclidean distance. This filtering approach and the distance of 250 m were used to optimize the number of spatially independent locations which did not allow an excessive reduction of the sample size (Figure S1). Furthermore, models were calculated only for those years with at least 15 occurrence records per target species (Table 1).

### 2.4 Environmental predictors

Species records and the environmental variables were incorporated into a geographic information system (GIS; software ArcMap 10.8) using the World Geodetic System 1984 (WGS84) and the Universal Transverse Mercator (UTM) 32 N projection. Based on the most common environmental predictors used to model fish distribution (Melo‐Merino et al., 2020) and the features of the study area, the following variables were used: water depth (m); seafloor slope (degree); seabed habitat type (SBH); mean sea surface temperature (SSTm;°C) and range (SSTr;°C); mean sea bottom temperature (SBTm;°C); chlorophyll concentration (Chl‐a; mg/m^3^); mean sea surface salinity (SSSm; practical salinity units—PSU); and mean dissolved oxygen (O_2_; mmol/m^3^).

Water depth was mapped using the points recorded every 30 s by the GPS during the surveys (La Manna et al., 2020, 2023); these points were used to create a raster bathymetry surface with a resolution of 250 m. Seafloor slope (the bathymetric gradient along the study area measured in degrees) was derived from water depth using “Slope” function in Spatial Analyst Tools in ArcGIS (resolution 250 m). Information about the seabed habitat types was derived from the European Marine Observation Data Network (EMODnet) Seabed Habitats project (Vasquez et al., 2021). Seabed habitat type (SBH) was a categorical variable, with nine classes, coded as follows: 1 = Mediterranean infralittoral rock; 2 = Coralligenous biocenosis; 3 = Mediterranean infralittoral coarse sediment; 4 = Mediterranean infralittoral sand; 5 = Biocenosis of Mediterranean muddy detritic bottoms; 6 = Mediterranean circalittoral coarse sediment; 7 = Biocenosis of Mediterranean open‐sea detritic bottoms on shelf‐edge; 8 = Biocenosis of *Posidonia oceanica*; and 9 = Facies of dead “mattes” of *Posidonia oceanica* without much epiflora. Daily sea surface temperature (SST) satellite data were obtained using the E.U. Copernicus Marine Service Information (Buongiorno et al., 2013). Then, the SST mean (SSTm) and range (SSTr, as difference between the maximum and the minimum SST) monthly raster maps at 0.01° (1 km) of spatial resolution were created using the plug‐in Marine Geospatial Ecology Tools 0.8a68 for ArcGIS (Roberts et al., 2010). The monthly mean sea bottom temperature (SBT), the monthly mean concentration of chlorophyll (Chl‐a), the monthly mean concentration of salinity (SSSm), and the monthly mean dissolved oxygen (O_2_) in seawater at 0.042° of resolution (ca. 4–5 km) were downloaded from the E.U. Copernicus Marine Service (Escudier et al., 2020, 2021; Nigam et al., 2021; Teruzzi et al., 2021). Then, SSTm, SSTr, SBT, SSSm, Chl‐a, and O_2_ raster surfaces were downscaled to the spatial resolution of 250 m using the “Kernel Interpolation with Barriers” tool in the Geostatistical Analyst Tools. Raster of the selected environmental predictors was built per each year (from 2019 to 2023) and for the whole period (2019–2023) using “Raster Calculator” function of Spatial Analyst Tool (Figure S2).

Before running the models, a correlation matrix of the Pearson correlation coefficients (*r*) between the environmental predictors was created using “Raster Correlations and Summary Statistics” tool in the SDM toolbox 2.5 (Brown et al., 2017) to evaluate the correlation between predictors: depth and O_2_ were not used in the models since the first was correlated with SBT and Chl‐a and the latter was correlated with Chl‐a (Figure S2).

### 2.5 Species distribution modeling

The species occurrence records and selected environmental variables were used to build SDMs by means of MaxEnt (MaxEnt version 3.4.1, Phillips et al., 2020). Even if many different methods have been used in SDM, such as traditional parametric and semi‐parametric regression models (e.g., GLM, GAM, and MARS) and machine learning methods (e.g., tree‐based models and support vector machine), MaxEnt is mostly used due to its highest performance when presence‐only data are available, the number of sightings is low, and spatial bias may occur due to unbalanced sampling (Ahmadi et al., 2023; Elith et al., 2006, 2011; Guisan et al., 2013; Valavi et al., 2022), as in the present study. Moreover, MaxEnt controls the complexity of the fitted functions according to the number of occurrence records (Elith et al., 2011), showing to perform better than other top‐performing SDMs (boosted regression trees and random forest) in case of low sample size (<30 records) (Valavi et al., 2022).

MaxEnt finds an optimum relation between environmental variables and species occurrence based on the maximum entropy algorithm (Elith et al., 2011) and produces as output a suitability index ranging from 0 (not suitable) to 1 (suitable). This suitability can be interpreted as the likelihood of presence within the environmental conditions used to build the model.

Per each species, the occurrence records were partitioned using 10‐fold cross‐validation, to assess the average behavior of the algorithms (Phillips et al., 2006), and the average of these repetitions was used as results. Furthermore, on the basis of the data limitations and the study area size, MaxEnt settings were chosen as follows: (1) auto‐feature class [as suggested by Phillips & Dudík, 2008 in case of small or biased datasets]; (2) regularization parameter equal to 1 to reduce overfitting; (3) all the available background points (5358) were used to increase the predictive performance of the model since the geographical extent of the models coincides with the area used by fishers (Phillips & Dudík, 2008); and (4) cloglog output, since it is most appropriate for estimating likelihood of presence (Phillips et al., 2020).

A jackknife analysis was used to estimate the contribution of each variable to the MaxEnt run. Furthermore, the AUC (area under the receiver operating characteristic curve) was used to estimate the model performance (Phillips et al., 2006). AUC values range between 0 and 1: models with AUC higher than 0.8 have more than good discrimination ability (Swets, 1988). In the end, the model robustness was evaluated by calculating the test AUC standard deviation (SD) and the difference between the train AUC values (using all presences) and the mean test AUC values: lower are the test AUC SD and the difference between the train AUC and mean test AUC values higher is the model robustness (Herkt et al., 2016).

In the end, using the cloglog output of MaxEnt (Phillips et al., 2020), for each species, we produced maps per each possible year and per the whole period (2019–2023); from the latter, the extensions (in km^2^) of the areas with habitat suitability higher than 0.6 within the SFCA, within the other part of the MPA, and outside the MPA boundaries were measured.

## 3 RESULTS

Of the initial 1538 occurrence records, 691 were used in the modeling exercises (Table 1). All MaxEnt models obtained AUC higher than 0.8 (with only two exceptions), indicating good‐to‐high accuracy in predicting species/taxa likelihood of presence (Table 2). Furthermore, in most models, the small difference between train AUC and mean test AUC values (Table 2) indicates the overall robustness and low overfitting of the models (Warren & Seifert, 2011). The percent contributions of each environmental variable to the model output per species/taxa in each investigated year and for the whole period (2019–2023) are shown in Table 2. SBH and slope were the variables almost always contributing to the habitat suitability, especially in the most resident species/taxa (e.g., *O. vulgaris* and *Scorpaena* spp.). Furthermore, the temperature‐related variables (SSTm, SSTr, and SBTm) also influenced the likelihood of the species presence (Table 2), but with a yearly changing pattern. Considering the whole period (2019–2023), SBTm influenced all species' presence, with the only exception being *Spondyliosoma cantharus* which was more influenced by SSTr. SSSm influenced the habitat suitability of *Phycis phycis* and *Spondyliosoma cantharus*, while the least important contributing variable was Chl‐a in all models (Figure S3).

**TABLE 2:** Percent contribution of the different variables to the MaxEnt models predicting the likelihood of species/taxa presence. Output refers to different years, depending on the number of available records, and the whole period (2019–2023).

Period	% Contribution							AUC	AUC_diff_
	SSTm	SSTr	SBTm	SSSm	Chl‐a	SBH	Slope	Mean ± SD	AUC_train_ ‐ AUC_test_
*Octopus vulgaris*									
2021	40.0	17.5	5.5	7.9	7.3	16.0	5.5	0.93 ± 0.06	0.03
2022	0.1	1.0	11.4	29.4	3.4	26.2	28.4	0.85 ± 0.14	0.07
2023	34.0	1.2	14.0	4.4	10.6	22.0	14.0	0.90 ± 0.05	0.04
2019–2023	2.8	18.0	30.0	7.6	3.2	23.0	15.0	0.89 ± 0.03	0.02
*Sepia officinalis*									
2019–2023	1.6	7.5	32.4	10.6	2.1	38.0	7.9	0.88 ± 0.04	0.03
*Diplodus* spp.									
2019	0.2	33.4	7.7	0.0	0.0	16.7	42.0	0.97 ± 0.02	0.01
2020	9.6	21.7	1.4	3.9	0.0	15.8	47.5	0.97 ± 0.01	0.02
2021	19.9	54.5	4.5	0.3	0.7	7.5	12.5	0.89 ± 0.07	0.05
2022	2.8	2.3	18.0	18.5	0.8	36.8	20.7	0.90 ± 0.18	0.05
2023	56.3	0.3	13.0	3.7	2.8	14.7	8.8	0.89 ± 0.04	0.03
2019–2023	1.8	15.3	24.4	7.3	2.2	18.4	30.6	0.89 ± 0.03	0.02
*Phycis phycis*									
2021	2.5	57.8	8.2	0.2	1.4	19.5	10.4	0.91 ± 0.05	0.04
2022	1.2	0.9	16.5	39.1	7.3	17.0	18.0	0.80 ± 0.11	0.10
2023	53.2	0.6	8.7	14.8	0.2	6.5	16.1	0.77 ± 0.08	0.11
2019–2023	0.4	6.1	14.3	49.6	1.6	4.6	23.5	0.81 ± 0.09	0.06
*Conger conger*									
2021	26.4	38.5	0.1	2.6	1.6	8.5	22.2	0.96 ± 0.02	0.02
2022	0.3	1.6	5.7	25.8	2.7	41.6	22.2	0.89 ± 0.14	0.06
2023	51.4	2.0	12.0	6.8	6.1	14.5	7.2	0.86 ± 0.05	0.05
2019–2023	0.9	14.5	20.3	13.1	2.7	13.5	35.1	0.89 ± 0.05	0.03
*Scorpaena* spp.									
2019	0.0	37.6	13.4	1.5	0.0	21.9	25.7	0.97 ± 0.49	0.01
2020	10.9	22.4	1.5	3.3	0.0	14.5	47.4	0.96 ± 0.09	0.02
2021	8.9	34.7	15.6	2.4	2.1	20.8	15.5	0.93 ± 0.04	0.01
2022	5.3	1.8	30.0	10.9	3.3	34.2	14.4	0.78 ± 0.12	0.09
2023	39.5	2.2	16.8	0.9	1.5	28.7	10.4	0.83 ± 0.07	0.05
2019–2023	2.2	9.5	24.3	8.8	3.2	26.0	26.1	0.85 ± 0.05	0.02
*Muraena helena*									
2021	16.2	38.2	0.9	1.7	0.4	13.6	29.0	0.96 ± 0.28	0.02
2023	66.3	0.4	16.3	1.8	0.8	8.3	6.1	0.80 ± 0.07	0.06
2019–2023	1.8	16.0	24.5	15.0	1.5	12.0	29.1	0.87 ± 0.05	0.02
*Spondyliosoma cantharus*									
2019	0.0	43.6	5.5	0.0	0.0	8.3	42.6	0.97 ± 0.80	0.01
2020	3.0	20.1	2.1	4.6	0.0	15.1	55.2	0.97 ± 0.28	0.02
2021	21.5	51.3	3.2	6.3	0.8	13.5	3.4	0.95 ± 0.03	0.01
2022	1.0	6.7	20.5	30.7	0.5	28.1	12.4	0.85 ± 0.07	0.07
2023	71.8	1.5	0.7	11.3	0.2	3.5	11.0	0.87 ± 0.04	0.07
2019–2023	0.9	26.4	15.5	22.1	2.7	11.1	21.3	0.90 ± 0.05	0.02

Abbreviations: Chl‐a, mean concentration of chlorophyll; SBH, seabed habitat type; SBTm, mean sea bottom temperature; SSSm, mean concentration of salinity; SSTm, mean sea surface temperature; SSTr, sea surface temperature range (difference between the maximum and the minimum SST).

**FIGURE 2:**
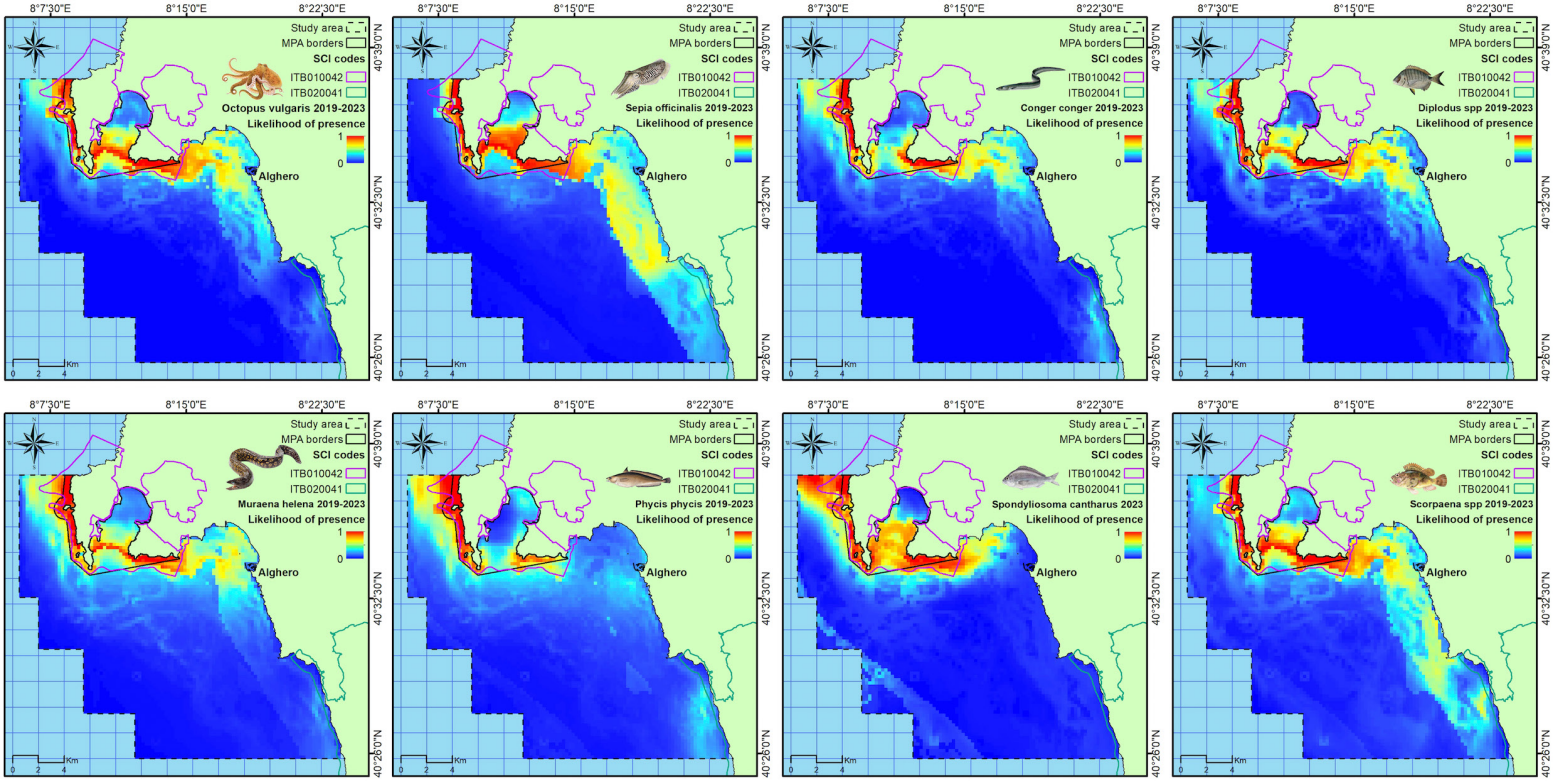
Likelihood of species/taxa presence as predicted by MaxEnt for the whole period (2019–2023).

The most suitable areas for the species/taxa change on an annual basis, but almost always overlap with some areas of the MPA (Figure 2, Figure S4).

Per each species/taxa, the largest surface area and relative percentage of the areas with habitat suitability higher than 0.6 were contained within the other areas of the MPA (excluding the SFCA), and the lowest within the SFCA and outside the MPA boundaries (Table 3).

**TABLE 3:** Surface area (in km^2^) and relative percentage of the areas with habitat suitability higher than 0.6 within the seasonal fishing closure area (SFCA), within the other parts of MPA (excluding the SFCA), within the MPA (whole), and outside the MPA boundaries per each species/taxa.

Species	SFCA		MPA (other than SFCA)		Whole MPA		Outside MPA	
	km^2^	%	km^2^	%	km^2^	%	km^2^	%
*Octopus vulgaris*	7.3	43	6.1	62	13.4	50	13.3	4
*Sepia officinalis*	7.9	47	8.0	82	15.9	60	10.6	3
*Diplodus* spp.	4.8	29	8.9	91	13.8	52	11.3	3
*Phycis phycis*	1.1	6	9.6	98	10.6	40	8.3	3
*Conger conger*	2.3	13	9.1	92	11.3	42	7.9	2
*Scorpaena* spp.	6.5	39	9.1	92	15.6	58	11.9	4
*Muraena helena*	3.5	21	9.3	95	12.8	48	9.8	3
*Spondyliosoma cantharus*	5.0	30	9.8	100	14.8	56	7.1	2

## 4 DISCUSSION

In the present study, we modeled the habitat suitability of commercial fish/taxa in the period between 2019 and 2023 based on several environmental predictors, using MaxEnt. The model outputs allow us to draw important insights about the spatial adequacy of the SFCA established within the MPA to protect some of the most exploited species by managing small‐scale fisheries. Furthermore, the study allowed understanding of local processes influencing species habitat selection and identification of essential areas for survival, reproduction, or feeding that could remain unrevealed in larger‐scale investigations (Puerta et al., 2015).

### 4.1 Model outputs

Temperature was a chief variable in predicting the habitat suitability of all species, while salinity resulted as an important predictor in the models of only two of them (*Phycis phycis* and *Spondyliosoma cantharus*). The distribution of all species responded similarly to the temperature environment: the likelihood of species presence increased with increasing SBT and decreased with increasing SSTm and SSTr. The role of temperature is not surprising since its relevancy in shaping marine biodiversity patterns (Tittensor et al., 2009) and driving shifts in geographic distributions has already been found in many fish and cephalopod species (D'Amen et al., 2023; Navarro et al., 2015; Pinsky et al., 2019; Poloczanska et al., 2016; Puerta et al., 2015). In fact, temperature may act directly at species physiological levels, or indirectly, affecting the availability of food resources and the consumption rates in the food webs (Puerta et al., 2015). Productivity‐related variables, such as Chl‐a, had no or negligible effect on the distribution of the studied species, likely because this variable influences the production and distribution of plankton, and in turn of planktivorous species, thus having little predictive potential in species with different feeding strategies (D'Amen et al., 2023). The fine‐spatial‐scale data on bathymetry, slope, and habitat type used in the models have also captured the spatial variation in the habitat suitability of the target species. In fact, slope and SBH were among the most important contributors in defining the likelihood of all species' presence. Namely most species preferred higher slope gradients and almost all habitat types, except for the infralittoral sand and the circalittoral coarse sediment. The high contribution of the type of seabed, in terms of slope and habitat, is linked to the ecological preference of the target species, which spend most of their life near the substrate, feeding on benthic macrofauna (see Table 1). Therefore, their likelihood of presence may depend on the presence and distribution of their preferred benthic prey as well as the need to hide from predators by finding suitable shelters (Katsanevakis et al., 2009). A high likelihood of species' presence was found mainly within the boundaries of the MPA, but also in the northern and deeper part of the study area for *Sepia officinalis* in the Gulf of Alghero and in the southernmost areas for *Scorpaena* spp.

The 34 models performed well, despite the low number of occurrence records for some species. AUC values indicated good‐to‐high model accuracy, coherently with the good performance of MaxEnt in modeling species distribution, especially at fine spatial scale (Elith et al., 2006; Moore et al., 2016). However, in the case of presence‐only distribution models, as MaxEnt, AUC cannot be considered a measure of model accuracy without potential bias, since it compares presences with background points (Gomez et al., 2015; Lobo et al., 2010), but it is generally accepted as a realistic method for model validation.

Being aware of model uncertainty and limitations is necessary, especially if the models are used in a management framework (Gomez et al., 2015). Thus, despite that the present modeling exercise is accurate and the model outputs are reliable, some relevant limitations of the study need to be highlighted. With the modeling approach here employed, we were able to highlight the current spatial distribution of potential habitats at the species/taxa level, but we could not consider the prediction of fish distribution at the population level. In fact, in addition to the environmental factors that influence the suitability of fish habitat, other population‐related factors (e.g., population size and density, age structure, behavior, predator avoidance, human exploitation) can substantially influence fish spatial distribution (Planque et al., 2011). Furthermore, fish distribution, that is, the association between fish species and habitat characteristics, depends on biotic and abiotic variables or a combination of both. However, biotic factors, which can provide insights into species and trophic interactions (Elith & Leathwick, 2009; Navarro et al., 2016), were not considered here. Since we cannot assume that the environmental variables are largely stronger than the biotic or the population‐related drivers (Planque et al., 2011), the results obtained can only give a partial representation of the species/taxa distribution. This limitation is especially relevant since biotic and population‐related drivers may be more influential at a local scale (Pearson & Dawson, 2003), like those used in the present study. Thus, these aspects deserve further investigation.

### 4.2 Management implications

Overall, our modeling exercise found that most species responded to the same combination of environmental variables and are mainly distributed within the same areas, highlighting the possibility of considering these target species as a single management unit that could be simultaneously protected within the MPA (Moore et al., 2016) with management effort such as the establishment of a fishing closure area. From 40% to 60% of the area with habitat suitability higher than 0.6 falls within the MPA boundaries. However, with a few exceptions (namely *O. vulgaris*, *S. officinalis*, and *Scorpaena* spp.), more than 70% of the most suitable areas were outside the SFCA. Therefore, if the management actions only took into consideration the protection of the most suitable areas for the distribution of the adult stages of the target species, the current spatial location of the SFCA could not be the most effective strategy. In fact, other areas, outside the SFCA but still within the MPA, could better protect the spatial traits of the investigated species. However, the SFCA, being a sheltered bay, could represent a nursery or a distribution area for the juvenile stages of many species. Furthermore, the seasonal closure could also be beneficial to protect some sensitive benthic habitats (such as the seagrass meadows of *Posidonia oceanica*) used by many species for shelter, food, or protection for their juveniles. These aspects were not considered here and should deserve further investigation to fully evaluate the spatial adequacy of the SFCA.

The modeling exercises also found that many areas outside the MPA are less suitable for the target species, likely because the seafloor is mostly composed of circalittoral coarse sediment, with almost no slope, which are conditions least favorable for the selected species. This result may highlight some socio‐economic implications. In fact, the seasonal closure of a portion of the MPA to small‐scale fisheries could force fishers to displace the fishing effort in areas less ecologically suitable for some of the target species, namely *O. vulgaris*, *S. officinalis*, and *Scorpaena* spp., for which about 40% of the most suitable areas are within the SFCA, with potential consequence on fishing profitability. The economic impact of the SFCA should therefore be considered together with the ecological benefits obtained within the closed area and the ecosystem costs (in terms of impact on habitat and benthic community) of the fishing effort redistribution in areas previously less exploited by small‐scale fisheries (Crear et al., 2021; Hiddink et al., 2006). In the end, we also found that the environmental space occupied by such species changes due to the response and adaptation to some variable conditions, such as those related to the thermal environment. In the Mediterranean Sea, environmental factors, such as temperature, show strong intra‐annual variation (Salat et al., 2002) resulting in species spatial pattern change on a yearly basis (Lloret‐Lloret et al., 2020). This result emphasizes the importance of regularly updating species occurrence data and their relationship with the present environmental conditions (Holsman et al., 2019). Furthermore, the future distribution of coastal fish species requires in‐depth investigation regarding predictions of how will be their habitat used under various climate change scenarios. This aspect, crucial for a comprehensive understanding of potential distribution shifts in marine ecosystems and thus to ensure sustainable fishing in the future and to assess the climate risk on the fishing communities (Bang et al., 2022; Chamberlain et al., 2023; Karp et al., 2022; Rogers et al., 2019), has not been explored in the present study and warrants further research.

## AUTHOR CONTRIBUTIONS

**G. La Manna:** Conceptualization (equal); data curation (equal); formal analysis (equal); writing – original draft (equal). **F. Ronchetti:** Data curation (equal); formal analysis (equal); writing – original draft (equal). **M. Moro Merella:** Investigation (equal); writing – original draft (equal). **R. Vargiu:** Investigation (equal); writing – original draft (equal). **F. Perretti:** Investigation (equal); writing – original draft (equal). **G. Ceccherelli:** Conceptualization (equal); writing – original draft (equal).

## FUNDING INFORMATION

This study was supported by the SPAMI Twinning Programme 2022–2023—ensuring effective management and evaluation of SPAMIs—coordinated by SPA/RAC under the cooperation agreement between UNEP/MAP Secretariat of the Barcelona Convention and the Italian Ministry of Environment and Energy Security. This study has also received funding from the European Union Next‐Generation EU (Piano Nazionale Di Ripresa E Resilienza (PNRR)—Missione 4 Componente 2, “Dalla ricerca all'impresa” Investimento 1.4 – D.D. 1034 17/06/2022, CN00000033). This manuscript reflects only the authors' views and opinions. Neither the European Union nor the European Commission can be considered responsible for them.

## CONFLICT OF INTEREST STATEMENT

None declared.

### OPEN RESEARCH BADGES

This article has earned an Open Data badge for making publicly available the digitally‐shareable data necessary to reproduce the reported results. The data is available at https://doi.org/10.5061/dryad.w3r2280zg.

## Supporting information


Figure S1.‐S4.

## Data Availability

The data that support the findings of this study are available from Gabriella La Manna here: https://datadryad.org/stash/share/‐NS6sR22HwOoecY2wZ9vk1BHPgnCVSkqykLAhvnb0Wc.
