# Supplementary material for: Using spatial distribution modeling of commercial species to inform management of small‐scale fisheries in a Mediterranean marine protected area
Source: Ecol Evol. 2024 Sep 13;14(9):e70169. doi: 10.1002/ece3.70169 (PMC11393775; doi:10.1002/ece3.70169)
Supplement: Supplementary file 1 — Figure S1.‐S4. [file ECE3-14-e70169-s001.docx]

**Spatial distribution modelling of commercial species informs management of small-scale fishery in a Mediterranean Marine Protected Area**

La Manna G.^1,2,3^, Ronchetti F.^3^, Moro Merella M.^1^, Vargiu R.^1^, Perretti F.^3^, Ceccherelli G. ^1,2,3^

**Supplementary Materials**

**Supplementary Material 1.** Maps showing the occurrence records of each species/taxa without spatial filtering (SF; in red) and after the spatial filtering (in green). The square boxes show the results of the spatial filtering with a zoom in a small portion of the study area.  **
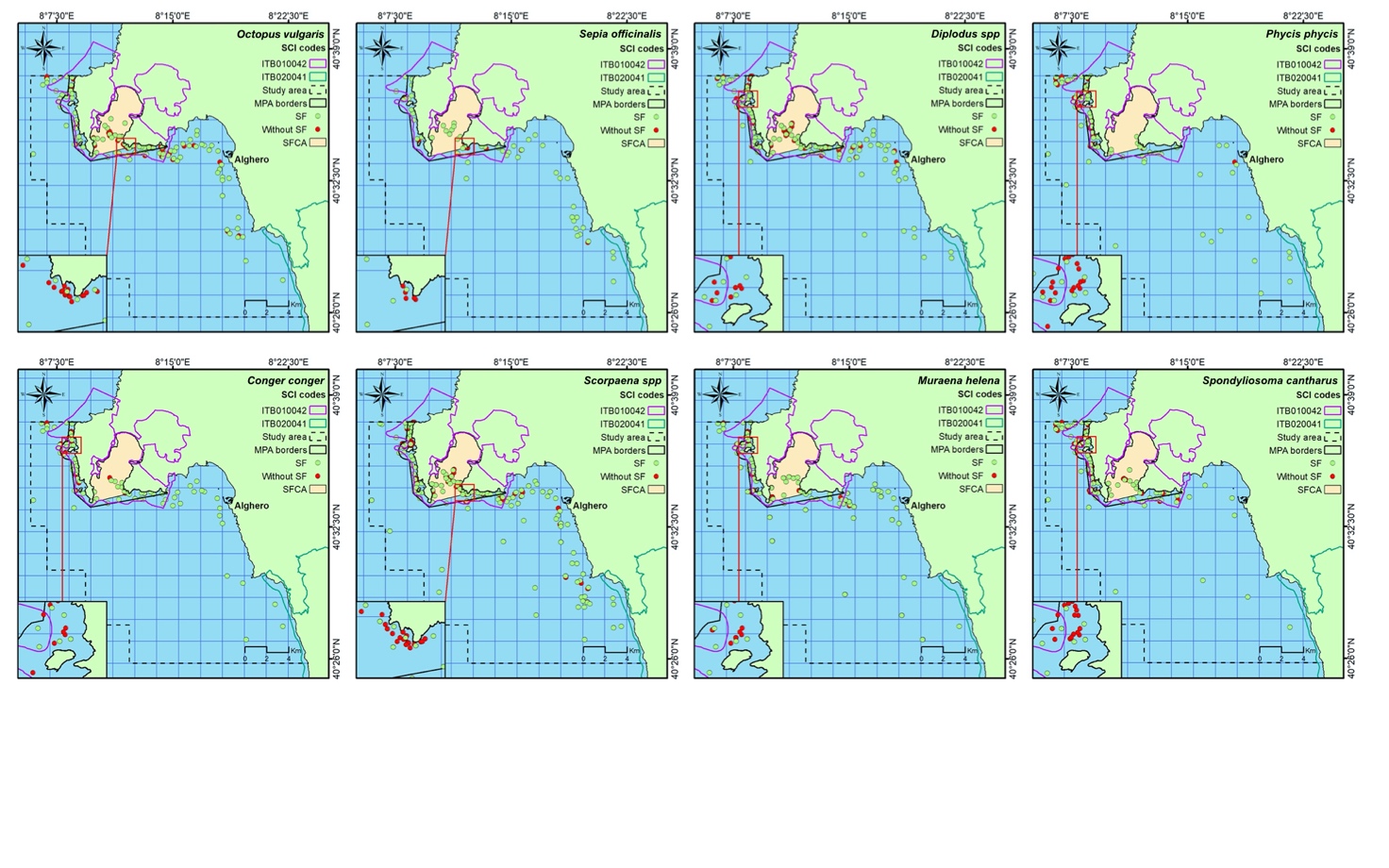
**

**Supplementary Material 2.** Environmental variables (SSTm, SSTr, SBT, Chl-a, SSS) used to build the MaxEnt models: A) 2019, B) 2020, C) 2021, D) 2022, E) 2023, F) 2019-2023 and G) constant predictors (Slope and SBH).

A)


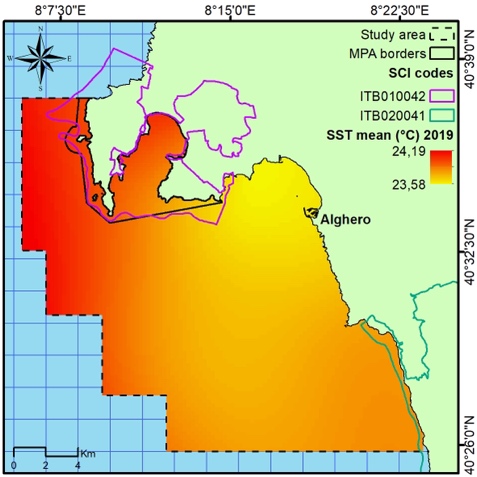

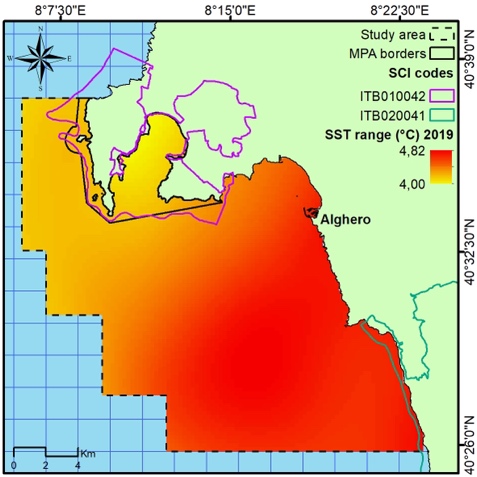

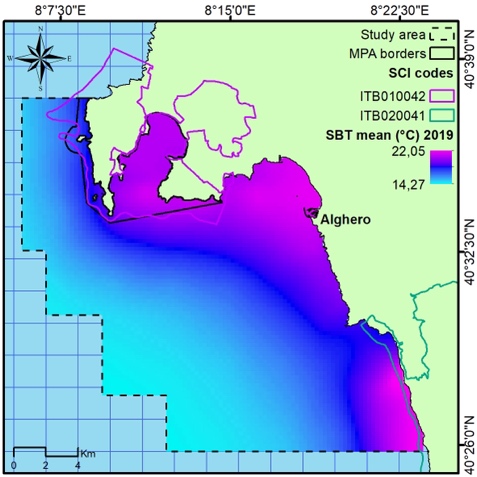

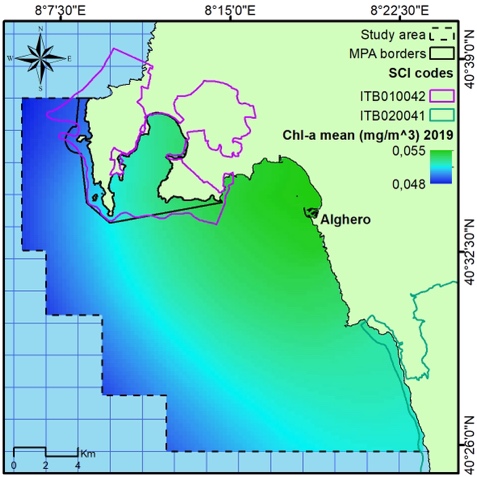

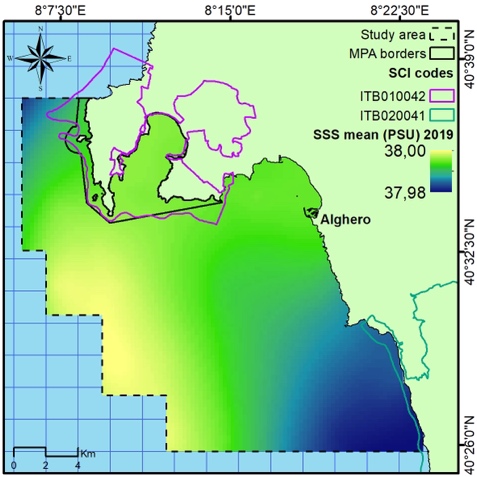


B)


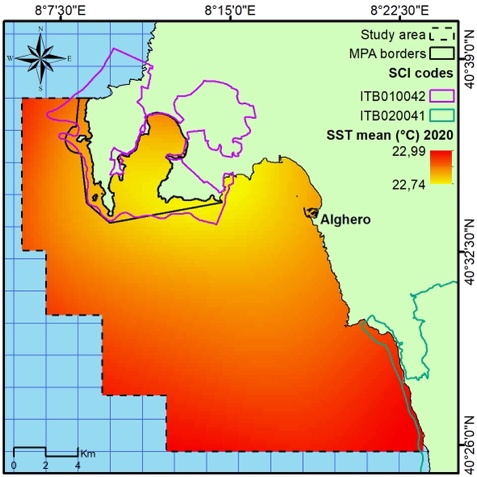

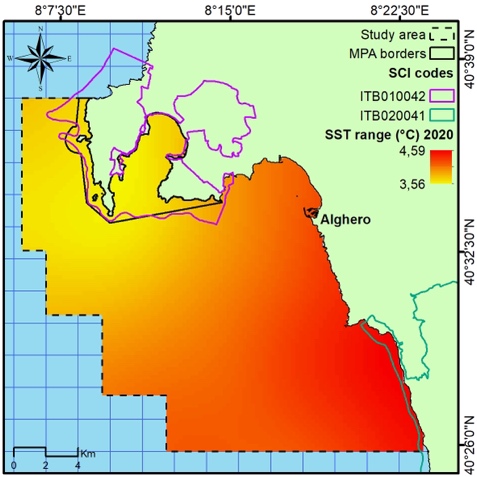

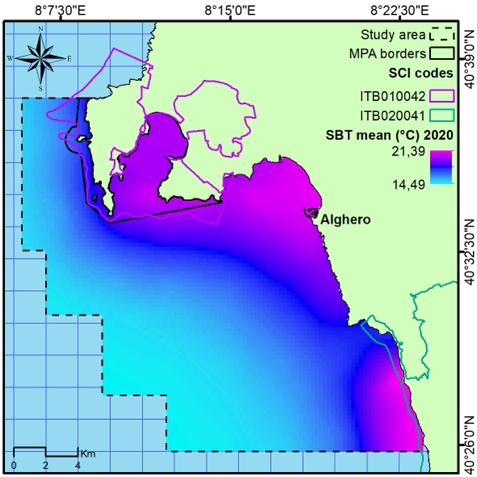

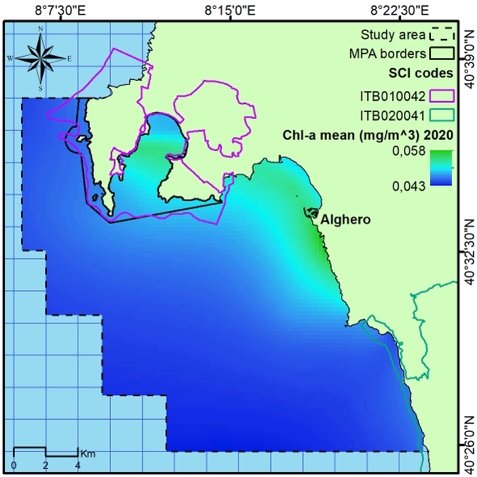

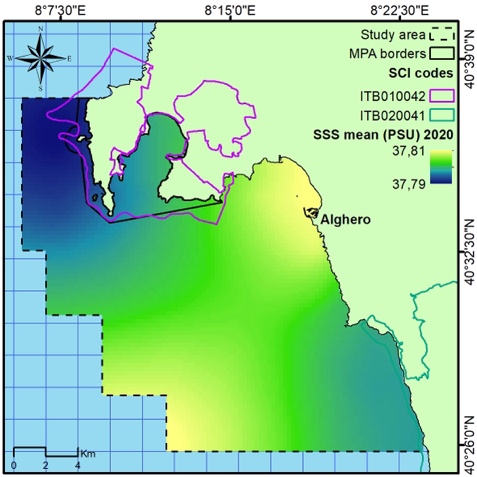


C)


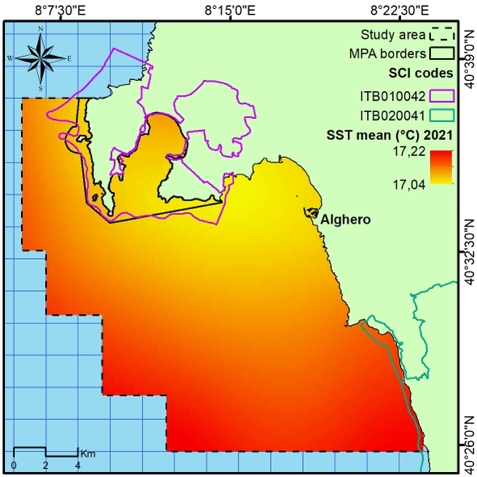

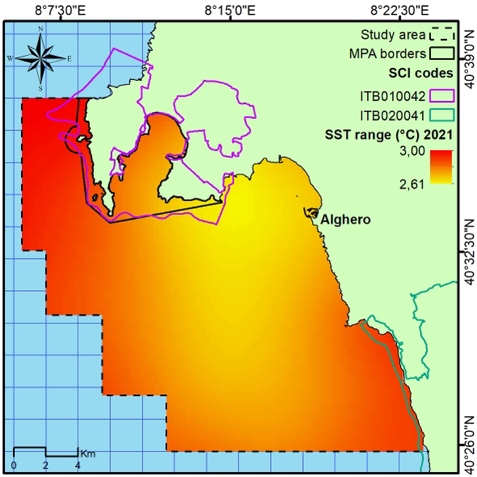

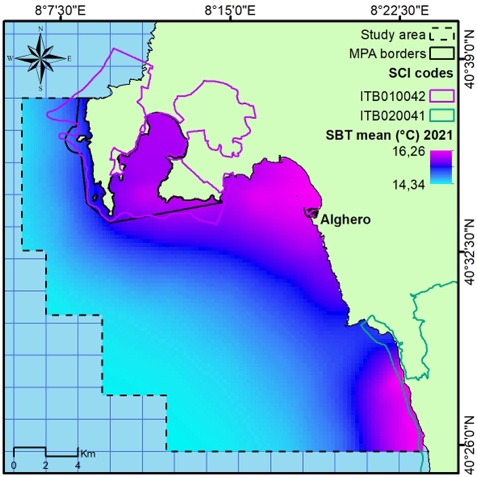

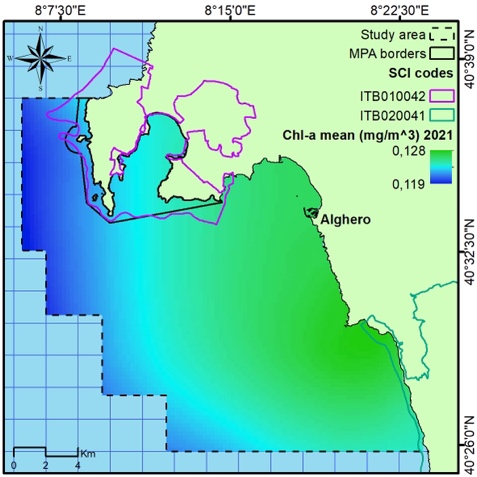

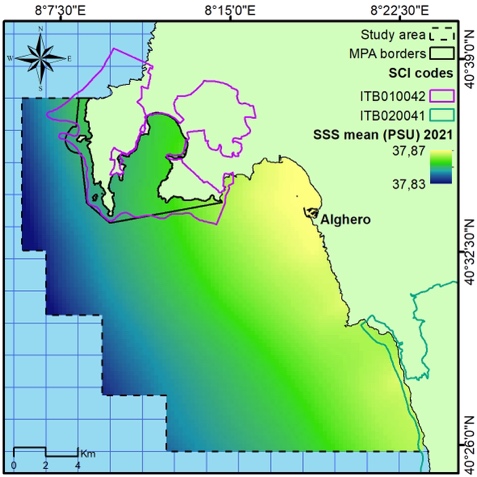


D)


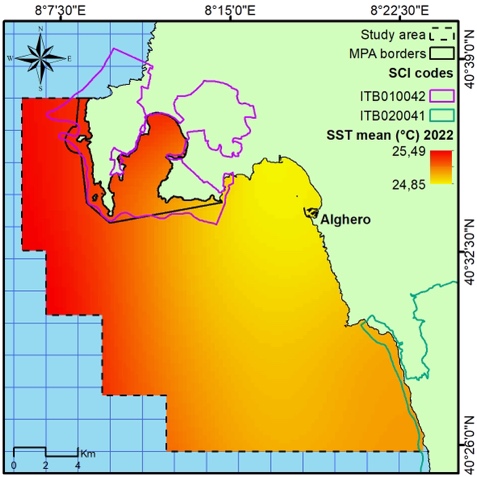

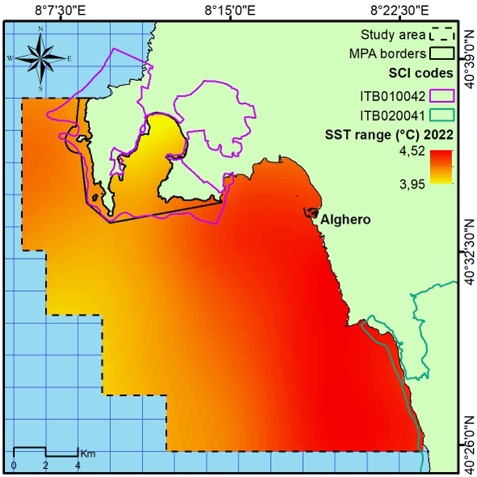

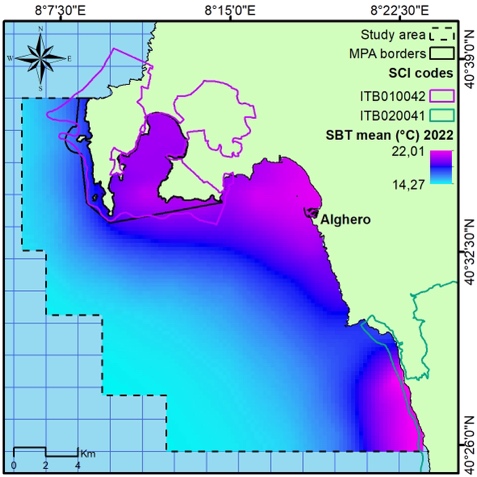

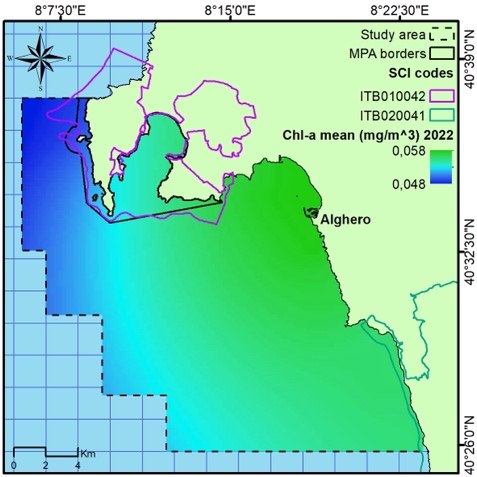

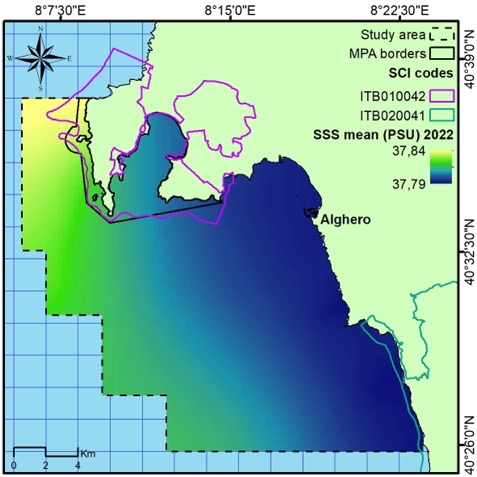


E)


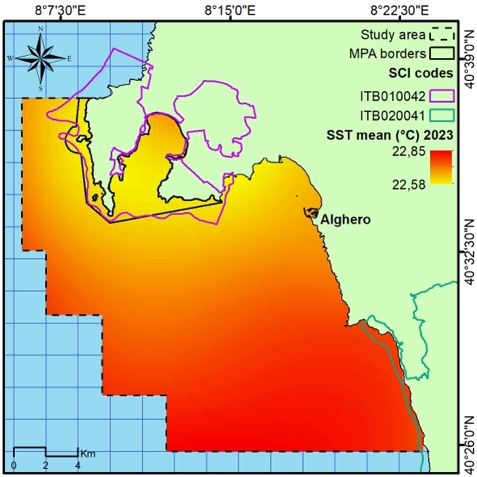

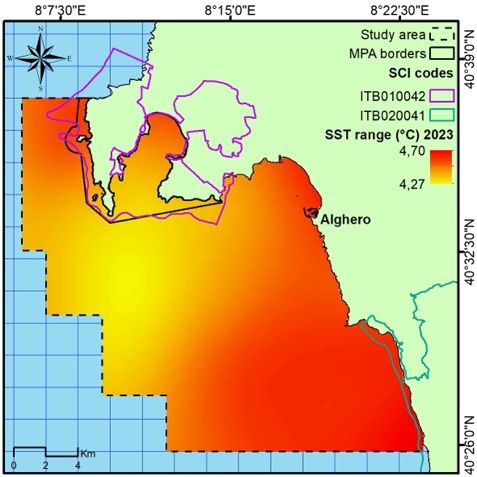

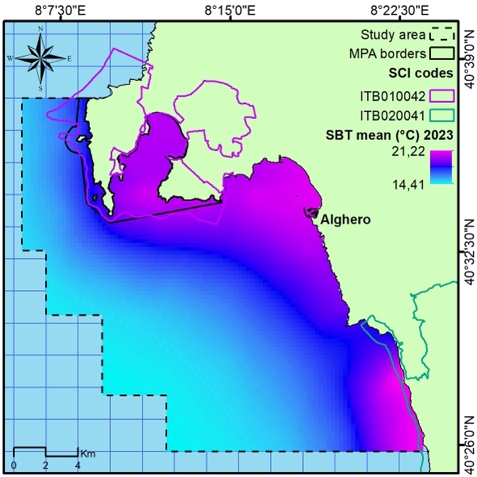

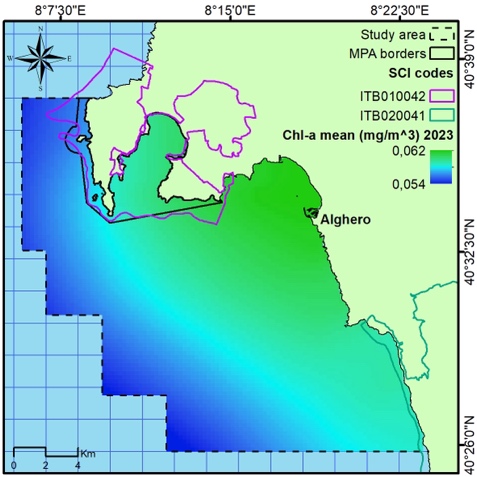

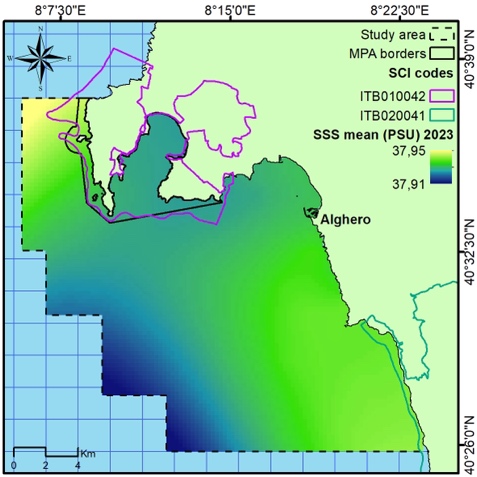


F)


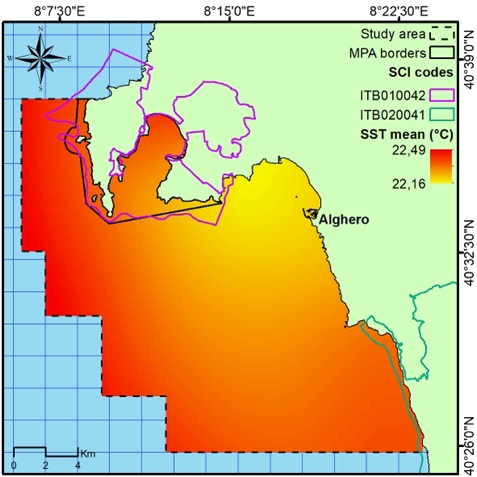

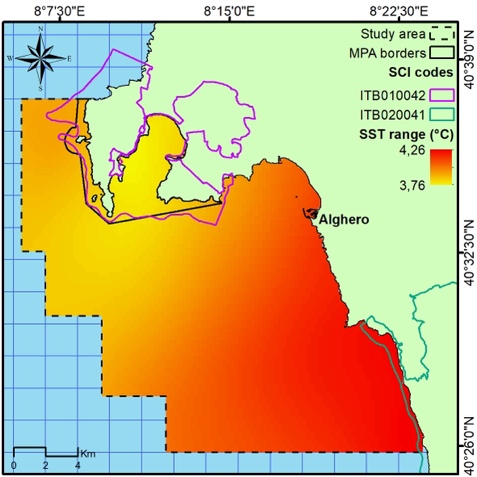

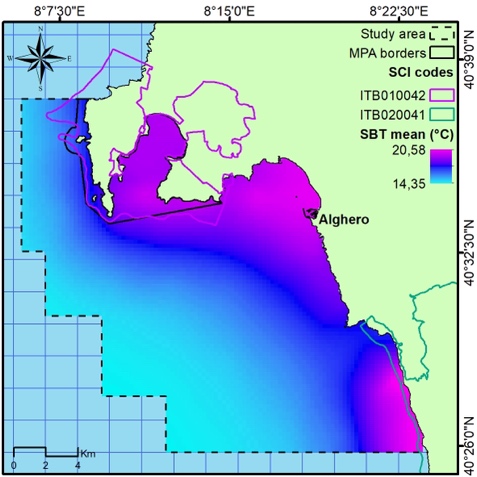

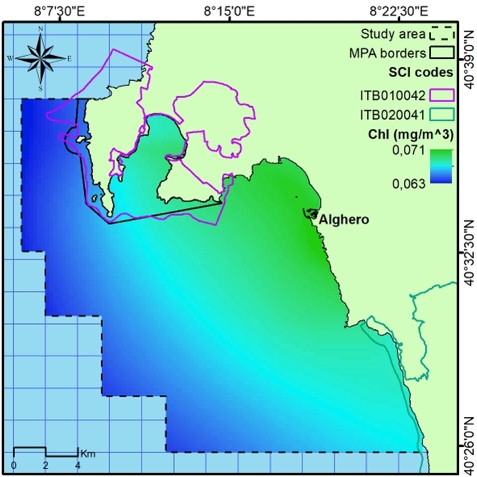

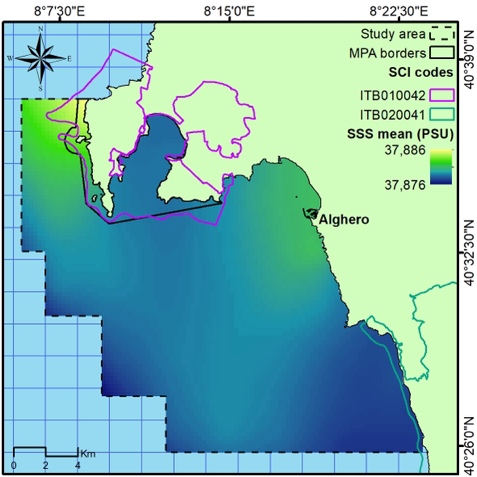


G)


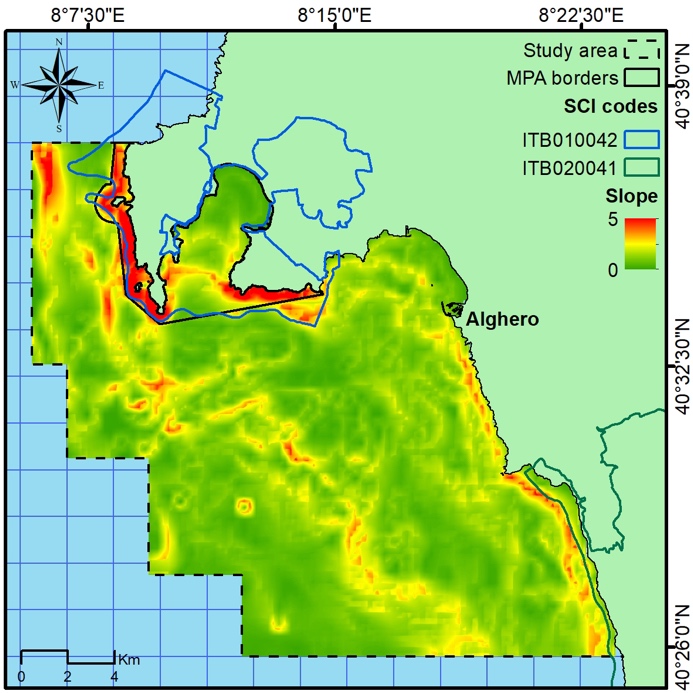

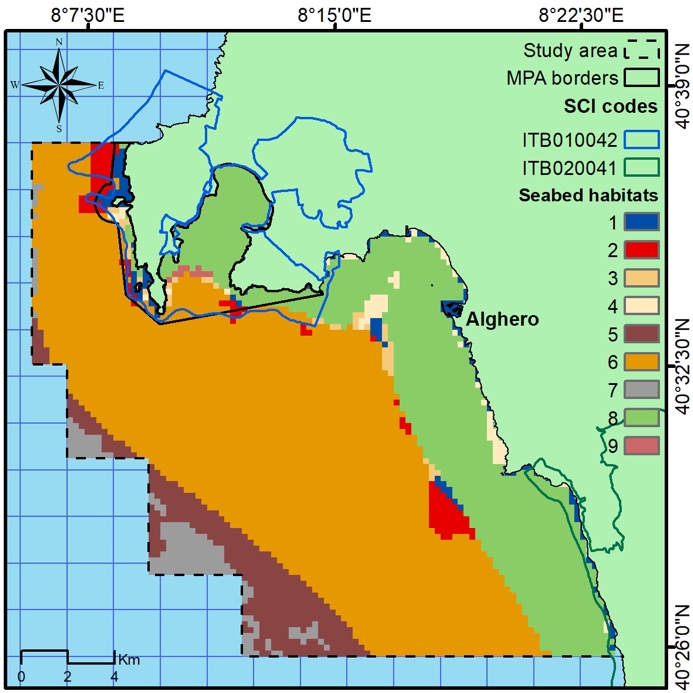


**Supplementary Material 3.** Effect of the main environmental variables on the likelihood of the species/taxa presence (cloglog output) for the whole period (2019-2023).

**
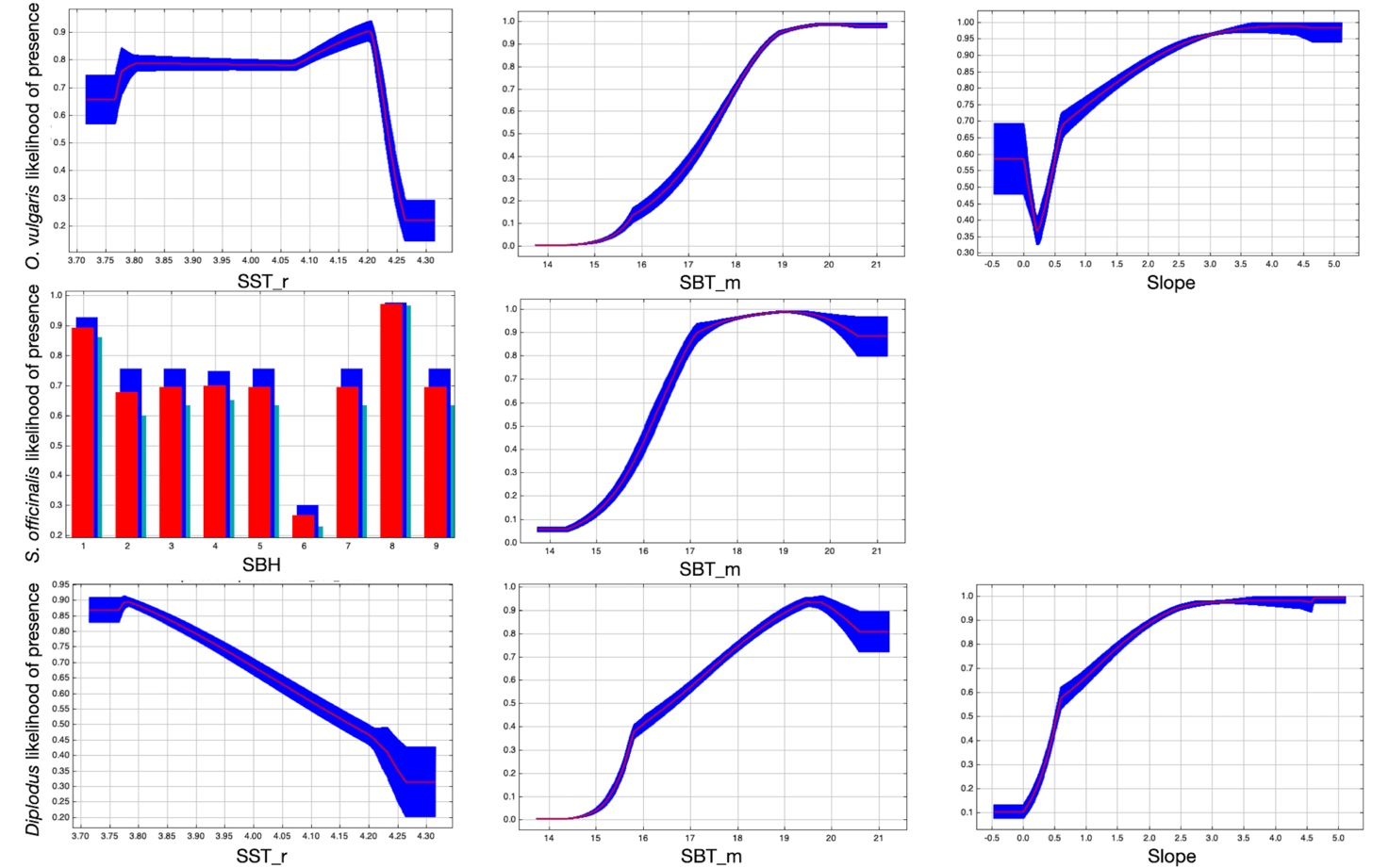
**

**
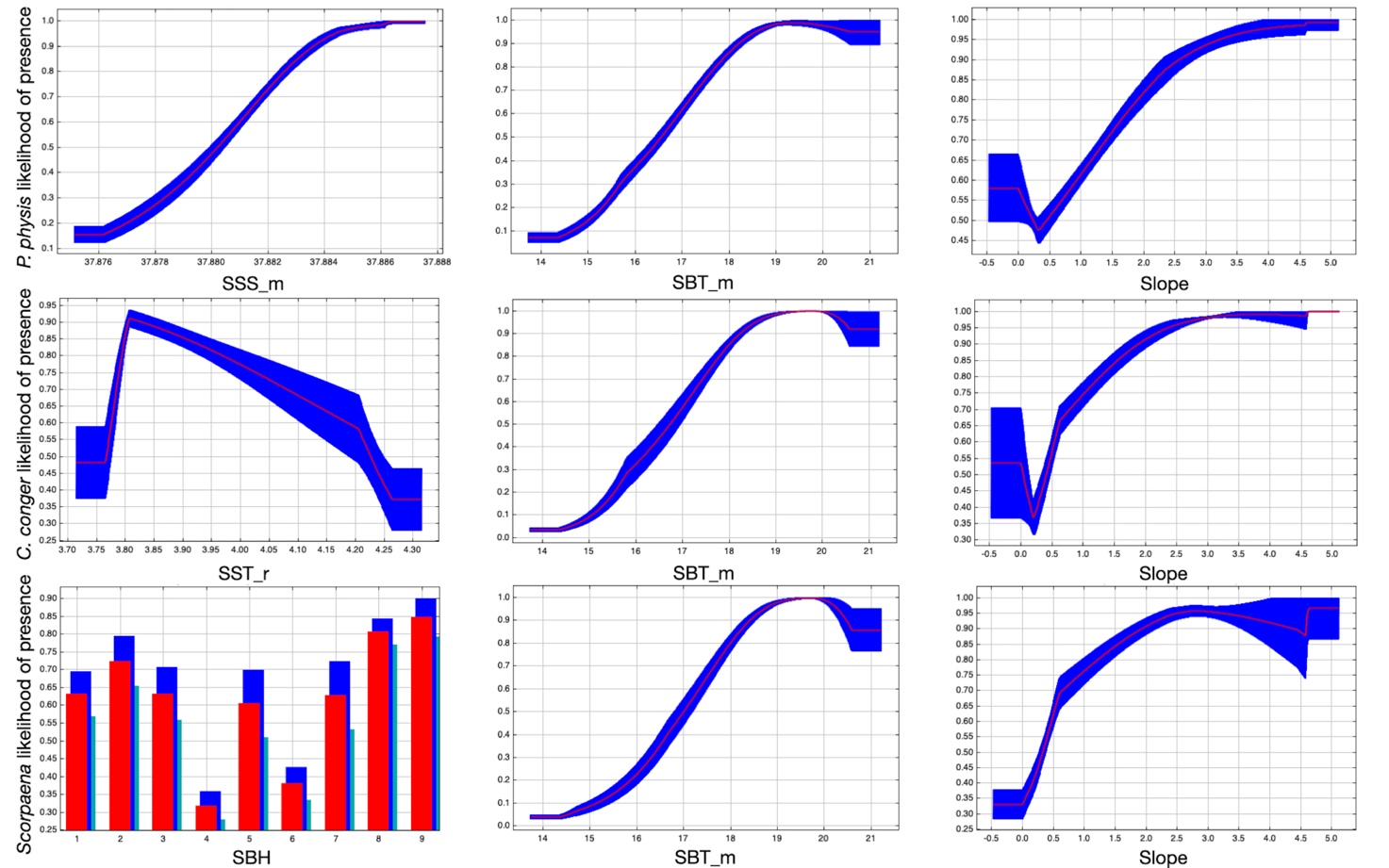
**

**
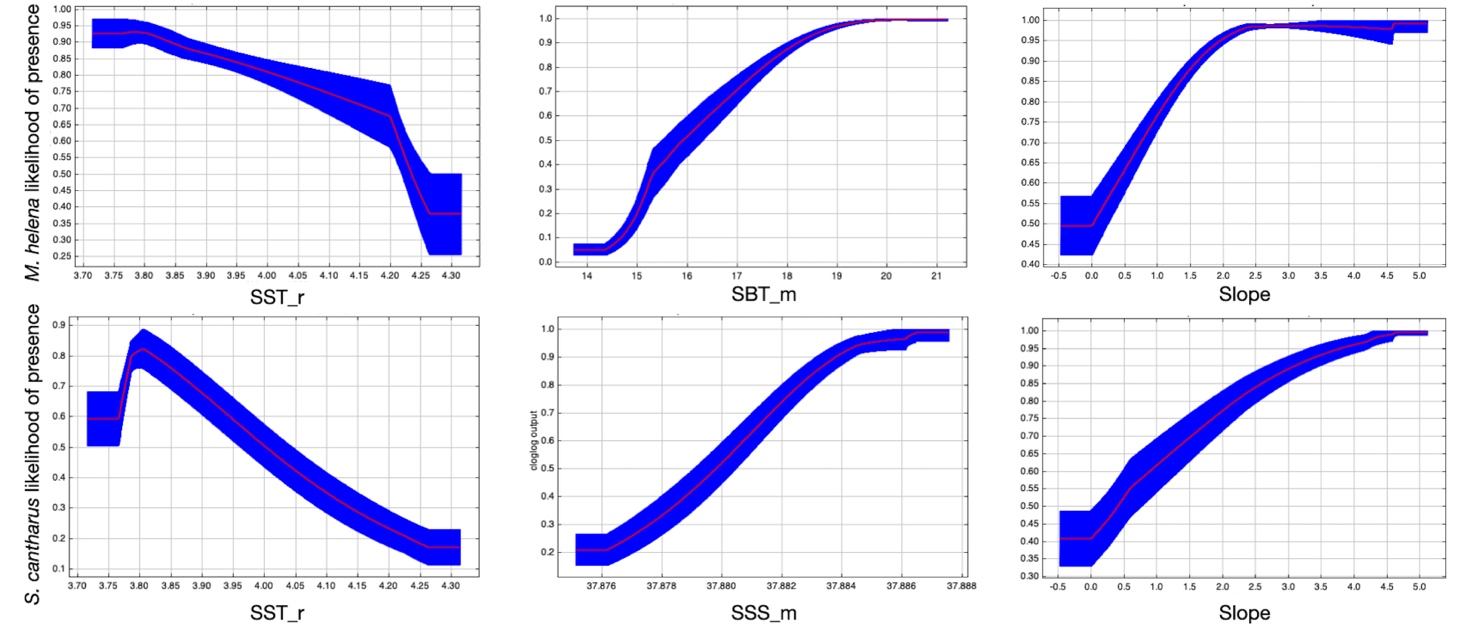
**

**Supplementary Material 4.**

1. Likelihood of *Scorpaena* spp. presence as predicted by MaxEnt in the years 2019, 2020, 2021, 2022, 2023.

**
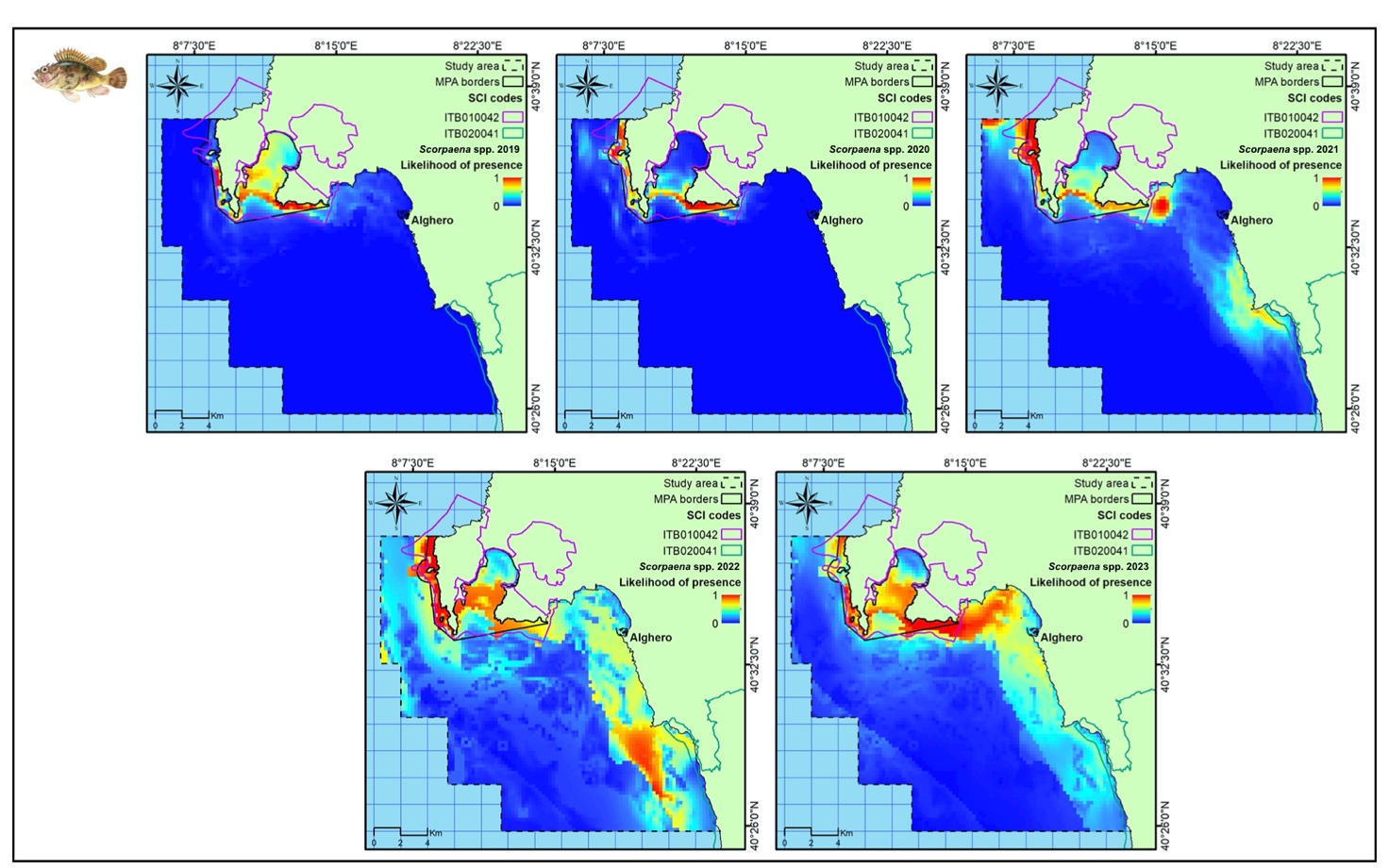
**

1. Likelihood of *Spondyliosoma cantharus* presence as predicted by MaxEnt in the years 2019, 2020, 2021, 2022, 2023.


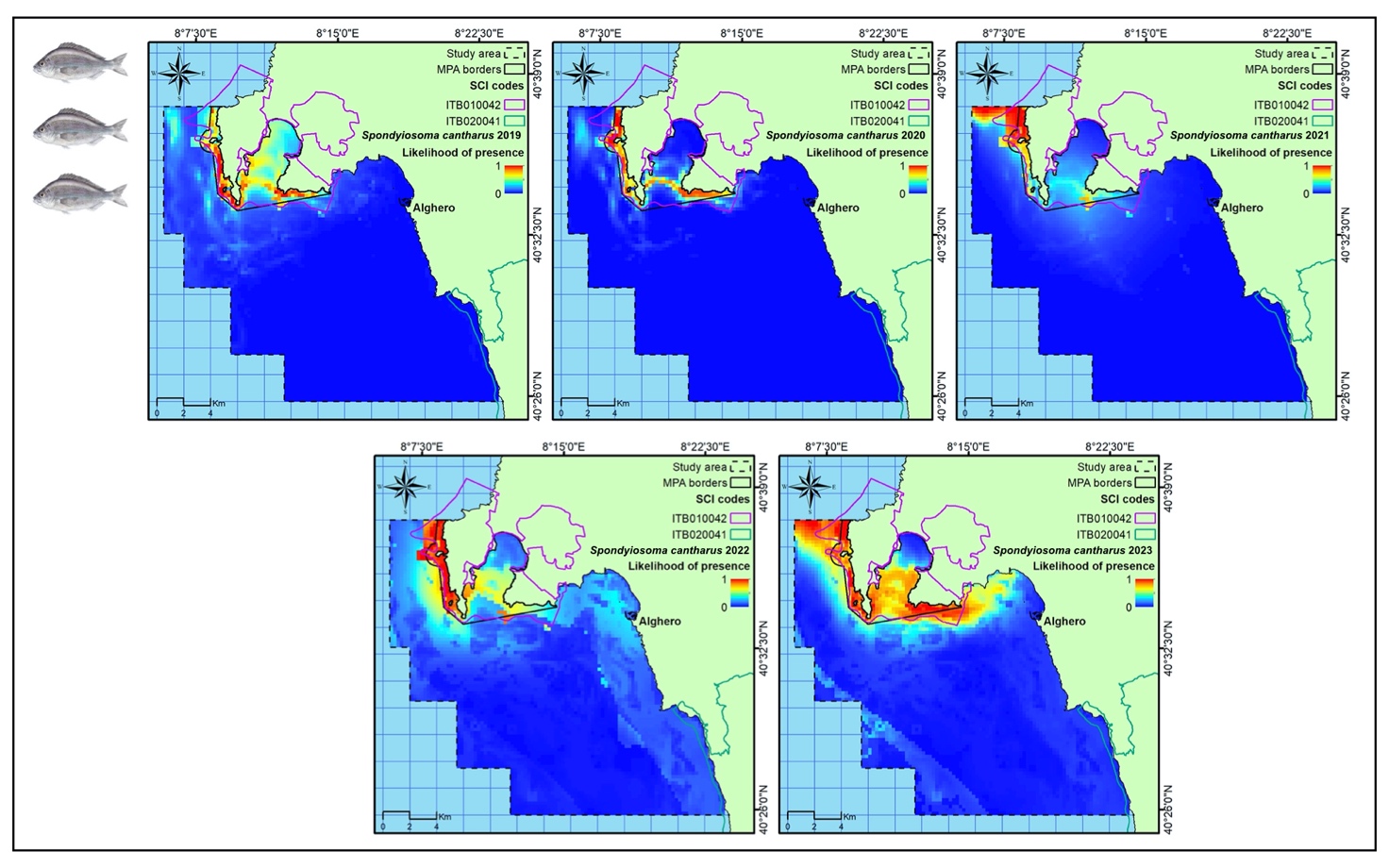


1. Likelihood of *Muraena* helena presence as predicted by MaxEnt in the years 2021 and 2023 and likelihood of *Phycis phycis* presence in the years 2021, 2022, 2023.

**
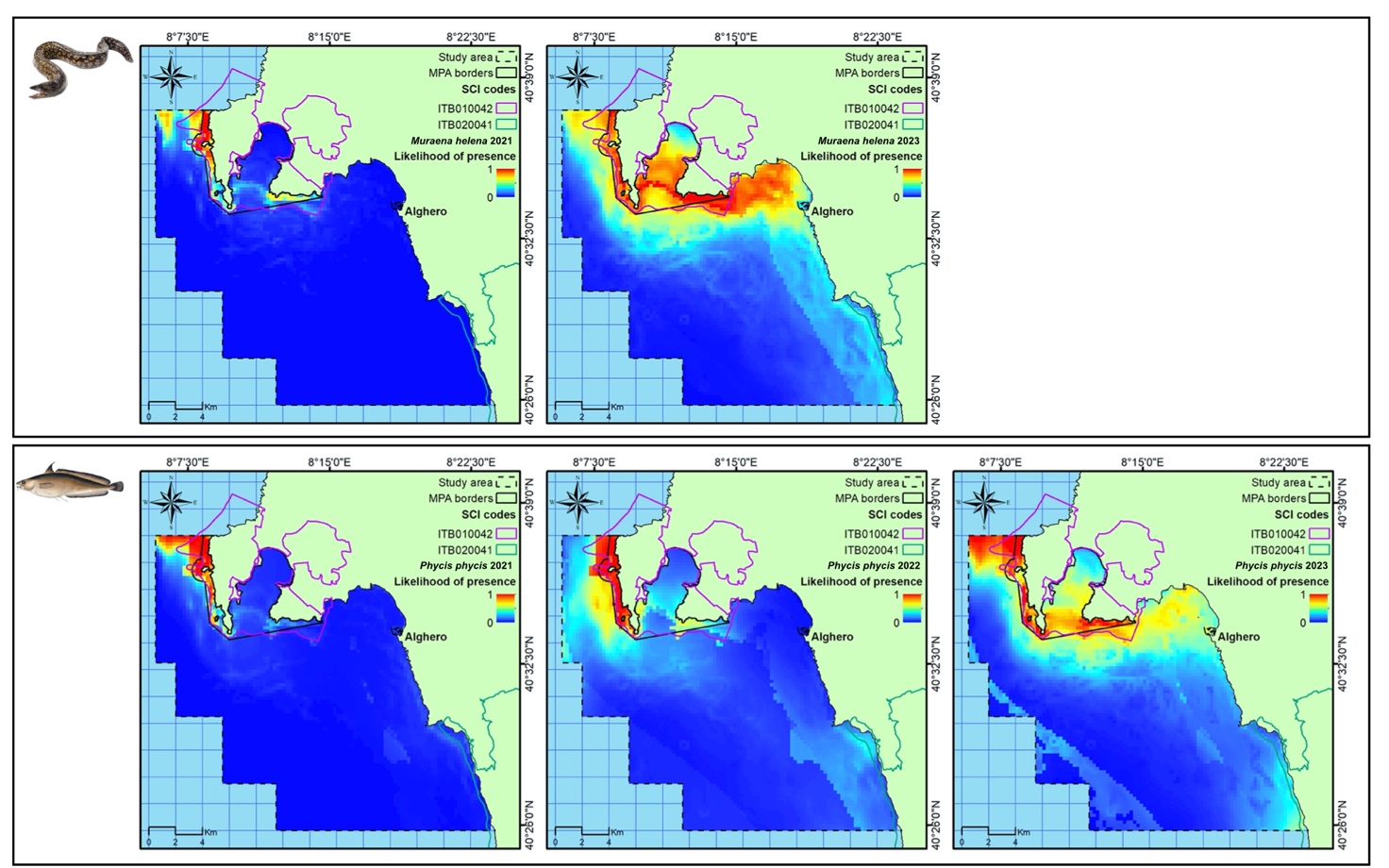
**

1. Likelihood of *Diplodus* spp. presence as predicted by MaxEnt in the years 2019, 2020, 2021, 2022 and 2023.

**
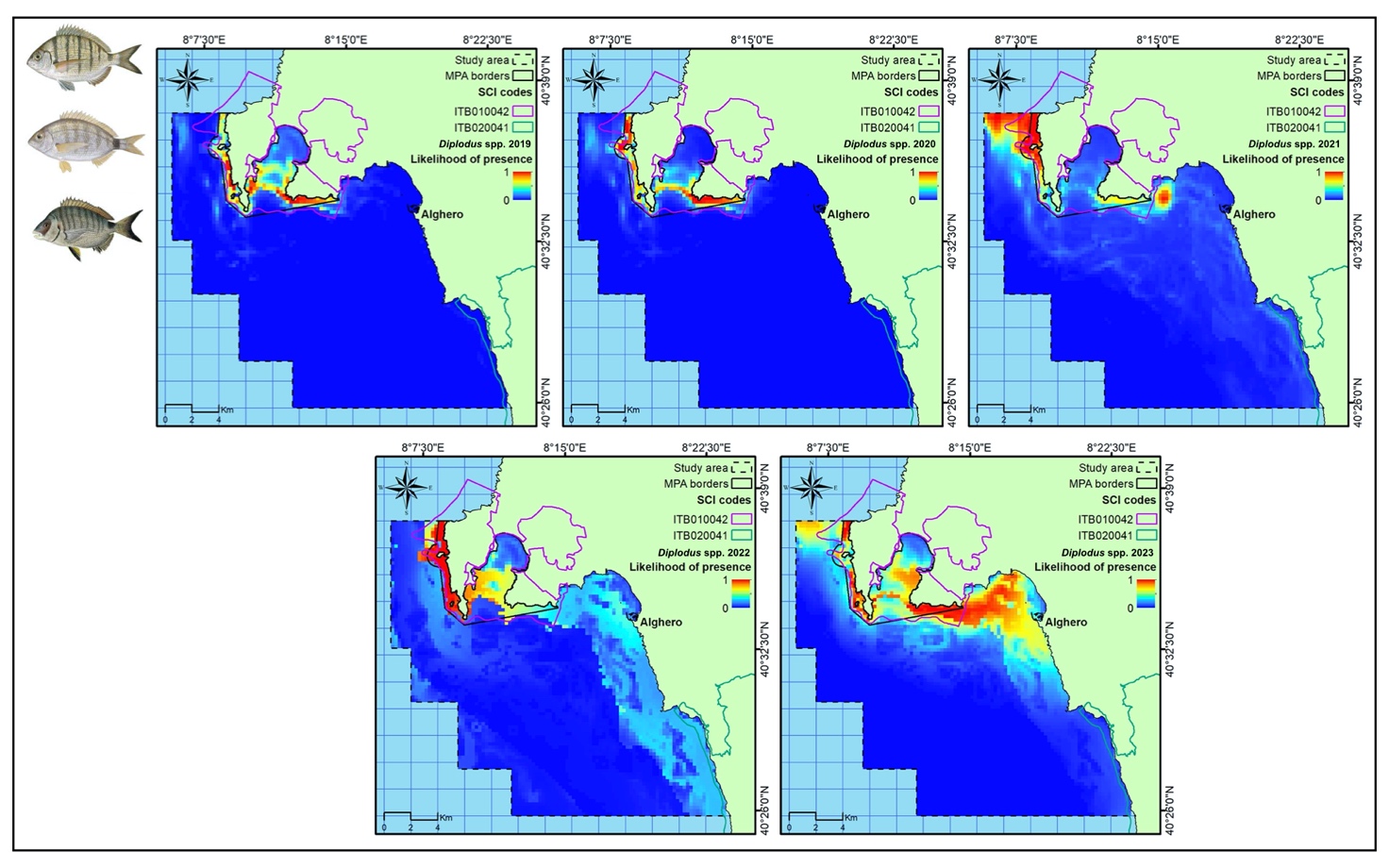
**

1. Likelihood of *Octopus vulgaris* presence as predicted by MaxEnt in the years 2021, 2022 and 2023 and likelihood of *Conger conger* presence in the years 2021, 2022, 2023.

**
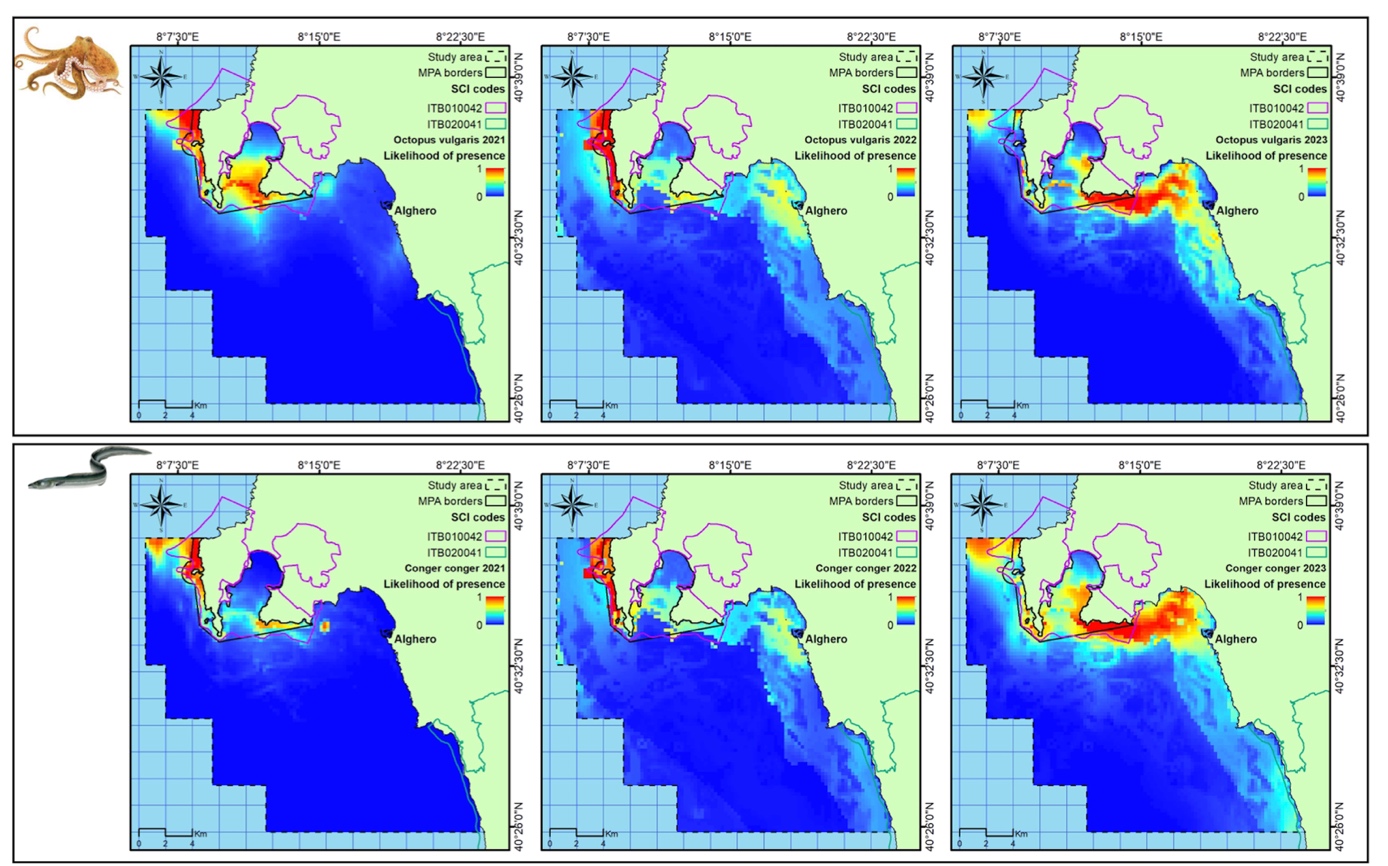
**
